# Multimodal Hand Hygiene Interventions and Clinical Healthcare-Associated Infection Outcomes in Acute Care Hospitals: A Systematic Review of Quasi-Experimental Studies

**DOI:** 10.3390/jcm15103882

**Published:** 2026-05-18

**Authors:** Emilia Doaga Pruna, Lavinia Davidescu, Maria Sorop-Florea, Ioan Demeter, Stela Iurciuc, Norberth-Istvan Varga, Vlad Laurentiu David, Florina Buleu, Florin George Horhat

**Affiliations:** 1 Doctoral School, “Victor Babes” University of Medicine and Pharmacy, Eftimie Murgu Square, No. 2, 300041 Timisoara, Romania; emilia.pruna@umft.ro; 2 Department of Medical Disciplines, Faculty of Medicine and Pharmacy, University of Oradea, 410073 Oradea, Romania; lavinia.davidescu@didactic.uoradea.ro; 3 “Constantin Brancusi” University, Tineretului Street, No. 4, 210185 Targu Jiu, Romania; maria.sorop@e-ucb.ro; 4 Department of Nursing, “Victor Babes” University of Medicine and Pharmacy, Eftimie Murgu Square, No. 2, 300041 Timisoara, Romania; demeter.ioan@gmail.com (I.D.); iurciuc.stela@umft.ro (S.I.); 5 Multidisciplinary Research Center on Antimicrobial Resistance (MULTI-REZ), “Victor Babes” University of Medicine and Pharmacy, Eftimie Murgu Square, No. 2, 300041 Timisoara, Romania; horhat.florin@umft.ro; 6 Department of Pediatric Surgery and Orthopedics, “Victor Babes” University of Medicine and Pharmacy, Eftimie Murgu Square, No. 2, 300041 Timisoara, Romania; david.vlad@umft.ro; 7 Department VI, Discipline of Internal Medicine and Ambulatory Care, Prevention and Cardiovascular Recovery, Faculty of Medicine, “Victor Babes” University of Medicine and Pharmacy, Eftimie Murgu Square, No. 2, 300041 Timisoara, Romania; florina.buleu@umft.ro; 8 Department of Microbiology, “Victor Babes” University of Medicine and Pharmacy, Eftimie Murgu Square, No. 2, 300041 Timisoara, Romania

**Keywords:** hand hygiene, hospital acquired infections, healthcare-associated infections, acute care hospitals, quasi-experimental studies

## Abstract

**Background/Objectives:** Hand hygiene is a cornerstone of infection prevention, yet the extent to which multimodal institutional hand hygiene interventions translate into measurable reductions in healthcare-associated infections (HAIs) remains uncertain. This systematic review aimed to evaluate the association between hospital-wide or multi-ward multimodal hand hygiene interventions and clinical HAI outcomes in acute care hospitals. **Methods:** A structured literature search was conducted in PubMed, Scopus, Embase, and Google Scholar using a combination of Medical Subject Headings (MeSH) and free-text terms related to hand hygiene, healthcare-associated infections, hospital settings, and intervention strategies. Eligible studies were quasi-experimental designs, including before–after, controlled before–after, and interrupted time-series studies, evaluating multimodal hand hygiene interventions implemented at hospital-wide or multi-ward level and reporting clinical HAI outcomes. Two reviewers independently assessed risk of bias using the ROBINS-I tool, and certainty of evidence across major outcome categories was summarized using GRADE. **Results:** twelve studies met the inclusion criteria. Overall, multimodal hand hygiene interventions were generally associated with favorable directional trends in clinical outcomes. Reductions were most consistent for broader institutional HAI measures and some device-associated infections, particularly central line-associated bloodstream infections. In contrast, organism-specific outcomes, including methicillin-resistant *Staphylococcus aureus*, vancomycin-resistant *Enterococcus*, and *Clostridioides difficile*, were more heterogeneous across studies and settings. All included studies were judged to be at serious or critical overall risk of bias, primarily because of confounding, lack of contemporaneous controls, co-interventions, and phased implementation. **Conclusions**: Multimodal hand hygiene programs in acute care hospitals may be associated with improvement in selected clinically relevant HAI outcomes, particularly at the institutional level. However, the overall certainty of evidence remains low to very low, and the strength of inference is limited by the non-randomized nature of the available studies and the difficulty of isolating the independent effect of hand hygiene within complex infection-prevention strategies.

## 1. Introduction

Healthcare-associated infections (HAIs) remain among the most frequent and consequential adverse events in modern hospital care. In the United States, a large multistate point-prevalence survey estimated that 4.0% of hospitalized patients had at least one HAI, corresponding to approximately 648,000 patients with 721,800 infections in acute care hospitals in a single year [1]. In Europe, the burden is similarly substantial: point-prevalence data from acute care hospitals across the European Union and European Economic Area estimated that 6.5% of patients had at least one HAI on any given day, with millions of patients affected annually [2]. Beyond prevalence alone, incidence-based modelling has shown that the cumulative population burden of common HAIs is extremely high, with a magnitude comparable to that of other major communicable diseases, reflecting their effects on mortality, morbidity, prolonged hospitalization, and healthcare resource use [3].

Because HAIs arise through multiple transmission pathways, their prevention requires a broad infection prevention and control framework. Established measures include hand hygiene, transmission-based precautions, environmental cleaning, and antimicrobial stewardship [4,5,6,7,8]. Among these, however, hand hygiene remains the most universally applicable and scalable intervention, because the hands of healthcare workers are the principal vehicle for cross-transmission of pathogens between patients and within the healthcare environment [5]. For this reason, major guidelines have consistently positioned hand hygiene as a cornerstone of HAI prevention and as a key component of safe patient care [4,5]. At the same time, hand hygiene is not a stand-alone solution, and its implementation must be understood within the wider ecosystem of hospital infection prevention strategies [6,7,8].

Despite longstanding guideline endorsement, hand hygiene performance remains inconsistent in routine practice. A major systematic review of compliance studies demonstrated wide variation in adherence across hospital settings and professional groups, with overall compliance often remaining suboptimal [9]. More recent reviews of intervention studies have similarly shown that the hand hygiene literature is highly heterogeneous, encompassing single-component and multimodal interventions, diverse healthcare worker populations, markedly different hospital settings, and a broad range of outcome measures [10,11,12]. Importantly, many studies focus primarily on hand hygiene compliance as a process measure, whereas others combine hand hygiene with broader quality improvement or infection prevention bundles, making cross-study comparison difficult and limiting the interpretability of findings at the institutional level [10,11,12].

This heterogeneity creates a persistent evidence gap. While there is broad agreement that improving hand hygiene is desirable, the more clinically relevant question is whether hospital-wide multimodal hand hygiene programs are associated with measurable reductions in HAIs in acute care hospitals. Existing syntheses have often pooled studies from highly specialized settings, such as intensive care units, combined diverse intervention models, or emphasized process outcomes over clinical infection endpoints [10,11,12]. In addition, when hand hygiene is embedded within broader infection-control bundles, the specific contribution of hand hygiene becomes difficult to isolate. As a result, the evidence base remains less clear than it appears when the focus is narrowed to institution-level or multi-ward multimodal hand hygiene interventions evaluated against clinically meaningful infection outcomes [10,11,12].

Accordingly, the objective of the present systematic review was to evaluate quasi-experimental studies of hospital-wide or multi-ward multimodal hand hygiene interventions in acute care hospitals and to synthesize their impact on clinical HAI outcomes. By focusing specifically on multimodal hand hygiene programs, quasi-experimental designs, and infection-related endpoints, this review aims to provide a more clinically interpretable assessment of the extent to which institutional hand hygiene interventions are associated with reductions in healthcare-associated infections.

## 2. Materials and Methods

### 2.1. Search Strategy

This systematic review was conducted in accordance with the Preferred Reporting Items for Systematic Reviews and Meta-Analyses (PRISMA) statement [13]. The PRISMA checklist can be found in the Supplementary Files. This research was prospectively registered in the International Prospective Register of Systematic Reviews (PROSPERO) under registration number CRD420261339769.

The review question was developed using the Population, Intervention, Comparator, Outcome (PICO) framework. The population of interest consisted of patients and healthcare workers in acute care hospitals. The intervention of interest was a hospital-wide or multi-ward multimodal hand hygiene intervention. The comparator was the pre-intervention period, standard practice, or a contemporaneous comparator group where applicable. The primary outcome was the occurrence of healthcare-associated infections (HAIs).

The search process was conducted in two stages. An initial structured literature search was performed in PubMed and Google Scholar. The PubMed strategy used MeSH terms and free-text keywords. Before final synthesis, the electronic search was updated and expanded by rerunning PubMed and searching Embase and Scopus.

Full reproducible Boolean strategies for the electronic search are provided in Supplementary File S1.

Google Scholar and backward reference screening were also used as supplementary search approaches to identify additional potentially relevant reports and citation variants.

The search strategy was structured around four main domains: (1) hand hygiene, (2) healthcare-associated infections, (3) acute care hospital setting, and (4) quasi-experimental intervention designs. The PubMed strategy combined Medical Subject Headings (MeSH) and free-text terms, whereas the Embase and Scopus strategies were adapted using database-specific title/abstract/keyword syntax. The main hand hygiene terms included “Hand Hygiene”, “Hand Disinfection”, “hand hygiene”, “hand washing”, “handwashing”, and “hand disinfection”. The infection-related terms included “Cross Infection,” “healthcare-associated infection,” “hospital-acquired infection”, “nosocomial infection”, and specific outcomes such as CLABSI, CAUTI, VAP, *Clostridioides difficile*, MRSA, and VRE. Study-design terms included “interrupted time series”, “before and after”, “before-after”, “controlled before-after”, “quasi-experimental”, and “pre-post”. Setting terms included “Hospitals”, “hospital”, and “acute care”. Terms related to nursing homes and long-term care were excluded.

No date restrictions were applied. Results were restricted to English-language reports.

In addition to the primary electronic search, backward snowballing was performed by screening the reference lists of relevant review articles and included studies to identify potentially eligible additional reports. Any study identified through snowballing was subjected to the same screening and eligibility criteria as studies retrieved through the electronic database search.

### 2.2. Study Selection

Study selection was carried out in two consecutive stages: an initial screening of titles and abstracts, followed by a full-text assessment of all records considered potentially eligible. At both stages, studies were evaluated independently by two reviewers according to the predefined eligibility criteria. Any disagreement was resolved through discussion and consensus. When necessary, a third reviewer was available to arbitrate unresolved discrepancies.

**Figure 1:**
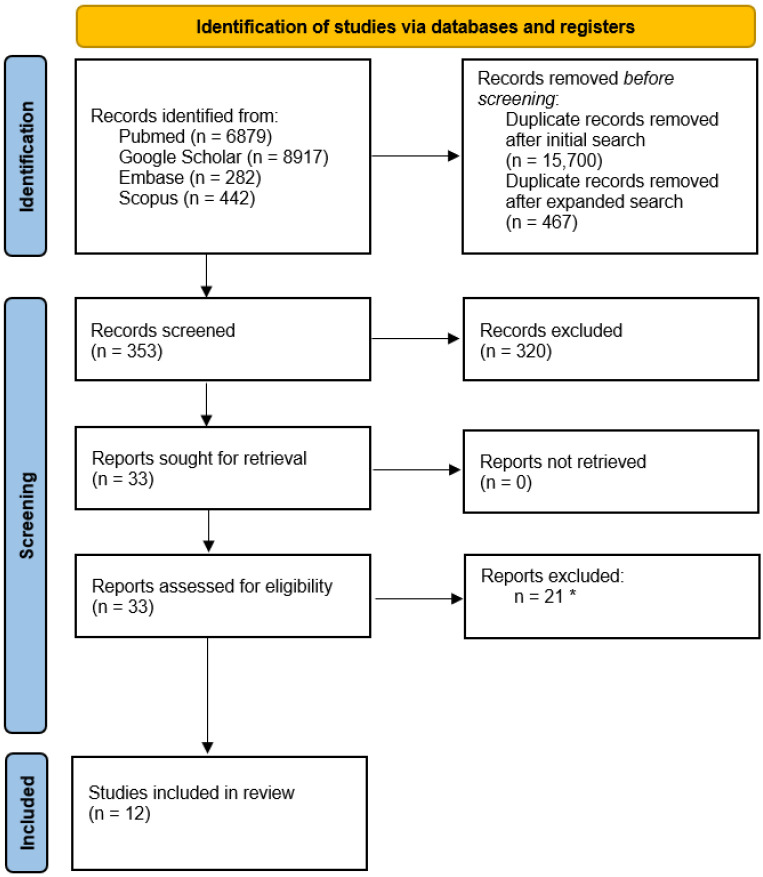
PRISMA Flowchart of Study Selection Strategy. * Reasons for exclusion are presented in Supplementary File S2.

All retrieved records were imported into Rayyan (Rayyan Systems, Inc., Cambridge, MA, USA) for duplicate detection. Duplicate citations were identified within Rayyan and manually checked before further screening. This two-step process was designed to ensure consistency in article selection and to minimize the risk of inappropriate exclusion of relevant studies. The study selection process is reported in the PRISMA flow diagram (see Figure 1), including the number of records identified, screened, excluded, assessed in full text, and included in the final qualitative synthesis.

#### 2.2.1. Eligibility Criteria

Studies were considered eligible if they were conducted in acute care hospital settings and evaluated a hand hygiene intervention implemented either hospital-wide or across multiple inpatient wards or departments. Only studies in which the intervention was multimodal were included, and eligible designs were restricted to quasi-experimental studies, including interrupted time series studies, controlled before-after studies, and uncontrolled before-after studies with clearly defined pre-intervention and post-intervention periods. In addition, studies were required to report at least one clinical outcome related to healthcare-associated infections.

Studies were excluded if they were conducted exclusively in long-term care facilities, nursing homes, outpatient settings, ambulatory care settings, or rehabilitation facilities. We also excluded studies performed exclusively in a single intensive care unit, neonatal intensive care unit, emergency department, or any other isolated single-unit setting. This decision was made a priori because these highly specialized environments differ substantially from general inpatient hospital settings in terms of patient case mix, baseline infection risk, intensity of care, and the frequent use of bundled preventive interventions, making comparison with hospital-level programs difficult and introducing considerable clinical heterogeneity.

Studies in which hand hygiene was not the clearly central intervention were excluded when it appeared only as one component of a broader infection prevention bundle (e.g., antimicrobial stewardship, screening, cohorting, isolation, or environmental decontamination) and the independent contribution of the hand hygiene programme could not reasonably be interpreted. By contrast, studies remained eligible when the principal intervention was a multimodal hand hygiene programme implemented at hospital-wide or multi-ward level, even if other background infection prevention activities or co-interventions were present, provided that hand hygiene promotion was the clearly identified focus of the intervention under evaluation. This approach was adopted because completely isolating hand hygiene from all other institutional infection prevention activity is rarely possible in real-world hospital implementation studies, whereas including studies in which hand hygiene was only a minor or inseparable component of a broader bundle would have introduced substantial confounding and limited causal interpretability.

Randomized controlled trials, including cluster-randomized trials, cross-sectional studies, purely descriptive observational studies, outbreak reports without evaluable intervention data, case series, case reports, editorials, letters, conference abstracts, and review articles were not eligible for inclusion. This restriction was applied because the review was specifically designed to evaluate hospital-wide or multi-ward multimodal hand hygiene programs as they are typically implemented in real-world acute care settings, where quasi-experimental designs are the most common evaluative approach. In such settings, individual randomization is often impractical and vulnerable to contamination, while even cluster-randomized designs address a somewhat different evaluative question from the one prespecified for this review. The exclusion of randomized studies therefore reflected the intended scope of the review rather than a judgment that such designs are methodologically uninformative.

#### 2.2.2. Definitions

For the purposes of this review, acute care hospitals were defined as institutions providing short-term inpatient medical and/or surgical care, including general hospitals, tertiary referral hospitals, and teaching hospitals. The term hospital-wide intervention was used to describe programs implemented across the institution as a whole, typically involving central leadership, hospital infection prevention structures, or institutional implementation strategies intended to influence practice broadly throughout inpatient care. A multi-ward intervention was defined as an intervention implemented in more than one inpatient ward or department and intended to influence routine clinical care across a substantial portion of the hospital, rather than within a single isolated clinical unit.

A multimodal hand hygiene intervention was defined as an intervention composed of at least two distinct hand hygiene improvement components. These could include education and training, reminders or posters, audit and feedback, increased availability of alcohol-based hand rub or soap, leadership involvement, local role models or champions, performance monitoring, or broader safety climate initiatives. For inclusion in this review, these hand hygiene components had to constitute the principal implementation focus of the programme being evaluated. Studies evaluating a single isolated measure alone, such as a poster campaign without any additional implementation component, a glove policy alone, a product comparison alone, or an electronic monitoring system without a broader improvement strategy, were not considered eligible unless that measure was clearly embedded within a larger multimodal institutional hand hygiene programme. Likewise, studies centred on broader infection prevention bundles were not considered eligible when hand hygiene promotion was not the clearly dominant intervention or when its independent contribution could not reasonably be interpreted.

Clinical outcomes were defined as outcomes reflecting the occurrence of healthcare-associated infections. Eligible outcomes included overall healthcare-associated infection rates, hospital-acquired infection rates, central line-associated bloodstream infection, catheter-associated urinary tract infection, ventilator-associated pneumonia or ventilator-associated events, *Clostridioides difficile* infection, methicillin-resistant *Staphylococcus aureus* (MRSA) acquisition or infection, vancomycin-resistant *Enterococcus* (VRE) acquisition or infection, and other clearly defined hospital-acquired infectious outcomes. By contrast, studies reporting only process measures, such as hand hygiene compliance rates or soap and alcohol-based hand rub consumption, were not considered sufficient for inclusion unless they were accompanied by clinical infection outcomes.

### 2.3. Data Analysis

Data extraction was performed using a standardized form developed specifically for this review. Two reviewers independently extracted data from each included study, and any discrepancies were resolved by discussion and agreement. The aim of the extraction process was to capture both the methodological characteristics of each study and the details necessary to interpret the intervention and its outcomes in a clinically meaningful way.

For each study, the following information was collected: the first author, year of publication, country, hospital setting, study design, number and type of included wards or departments, description of the intervention, duration of baseline and follow-up periods, comparator characteristics, healthcare worker groups involved, definitions of infection outcomes, denominators used for outcome measurement, main study findings, and statistical methods. Relevant secondary outcomes, such as hand hygiene compliance, were also recorded when available, as were major co-interventions and limitations noted by the authors.

Because substantial heterogeneity was anticipated across the included studies, a quantitative meta-analysis was not planned a priori. This heterogeneity was not limited to differences in intervention composition and hospital context, but also included marked variation in outcome definitions, denominators, surveillance methods, follow-up duration, study designs, and statistical approaches. In addition, the included studies reported conceptually related but non-commensurable outcomes, including overall HAI rates, device-associated infection rates, organism-specific acquisition or infection outcomes, and composite institutional indices, often using different units of measurement such as patient-days, device-days, admissions, exposure-days, or standardized infection ratios. For these reasons, statistical pooling was considered methodologically inappropriate, and formal summary heterogeneity statistics such as I^2^ were not calculated. The included studies were therefore analyzed descriptively and compared across several dimensions, including study design, intervention scope, intervention type, setting, clinical outcome category, duration of follow-up, and direction of effect. Particular emphasis was placed on whether interventions were associated with reductions in clearly defined clinical infection outcomes. Although process measures such as hand hygiene compliance were extracted where available, they were treated as secondary findings rather than primary evidence of intervention effectiveness.

### 2.4. Risk of Bias

The methodological quality of all included studies was assessed using the Risk Of Bias In Non-randomized Studies of Interventions (ROBINS-I) tool. This instrument was selected because all studies eligible for the review were non-randomized intervention studies and therefore required a structured appraisal method tailored to quasi-experimental designs.

Risk of bias assessment was conducted independently by two reviewers. Each study was evaluated across the standard ROBINS-I domains, including bias due to confounding, bias in selection of participants, bias in classification of interventions, bias due to deviations from intended interventions, bias due to missing data, bias in measurement of outcomes, and bias in selection of the reported result. After domain-level assessment, an overall judgment of risk of bias was assigned to each study. Disagreements between reviewers were resolved through discussion and consensus.

In addition, the overall certainty of evidence across major outcome categories was assessed using the GRADE approach.

### 2.5. Use of AI Tools

Rayyan (Rayyan Systems, Inc., Cambridge, MA, USA) was used during the study selection process for reference management, duplicate detection, and screening workflow support. In addition, ChatGPT version 5.1 (OpenAI, San Francisco, CA, USA) was used only for English-language editing and grammatical refinement after translation of the manuscript. No generative artificial intelligence tool was used for study selection decisions, data extraction, risk-of-bias assessment, evidence synthesis, interpretation of findings, or formulation of conclusions. All methodological decisions, data curation, analyses, synthesis, critical interpretation, and final manuscript content were performed, reviewed, and approved by the authors, who take full responsibility for the integrity and accuracy of the work.

## 3. Results

### 3.1. Overview of Included Studies

A total of 16,520 records were identified through the initial database search, including 6879 from PubMed and 8917 from Google Scholar. After removal of 15,700 duplicates, 96 records underwent title and abstract screening. The electronic search was then updated and expanded by adding Embase and Scopus. This updated search identified 724 additional records. After removal of 467 duplicates, 257 additional records underwent title and abstract screening. Out of 353 original articles screened at this stage, 320 were excluded based on our exclusion criteria, leaving thirty-three full-text articles to be assessed for eligibility. Twenty-one articles [14,15,16,17,18,19,20,21,22,23,24,25,26,27,28,29,30,31,32,33,34] were excluded after full-text review for reasons detailed in Supplementary File S2, leaving 12 studies [35,36,37,38,39,40,41,42,43,44,45,46] for inclusion in the qualitative synthesis.

The PRISMA workflow is illustrated in Figure 1.

Table 1 presents a detailed overview of the included studies. Overall, the evidence base was dominated by non-randomized interventional studies conducted in acute-care settings. Eight studies were single-centre before-after, quasi-experimental, or interrupted time-series evaluations, two used contemporaneous hospital comparators, one was a system-wide quality improvement evaluation across 11 hospitals, and one was a national ecological interrupted time-series study. The included studies were geographically diverse but weighted toward the United States (n = 6), with additional studies from Spain (n = 2), England and Wales, Saudi Arabia, Taiwan, and Vietnam. Interventions were overwhelmingly multimodal and most commonly combined education and training, improved availability or accessibility of alcohol-based hand rub, reminders or promotional materials, audit and feedback, and explicit leadership or accountability components; several studies further emphasized broader organisational change or process-improvement methods, including organisational climate interventions, continuous quality improvement tools with statistical process control, the Targeted Solutions Tool/DMAIC framework, and automated monitoring combined with supplementary promotional activities.

Clinical outcomes were heterogeneous and included overall healthcare-associated infection rates, organism-specific outcomes such as MRSA, VRE, or other antimicrobial-resistant organisms, device-associated infections such as CLABSI, CAUTI, and VAP, and, in the obstetrics/gynaecology setting, procedure-specific infections such as surgical site infection and endometritis. Study duration also varied substantially, ranging from short-term evaluations of several months to long-term campaigns extending up to nine years, which likely contributed to the marked clinical and methodological heterogeneity across the included literature.

### 3.2. Effects of Multimodal Hand Hygiene Interventions

Across the 12 included studies, multimodal hand hygiene interventions were generally associated with favorable trends in clinical healthcare-associated infection outcomes, although the magnitude and consistency of these effects varied substantially across settings, study designs, intervention components, and outcome definitions. The findings in this section are presented in as a structured narrative synthesis, and a summary of the main clinical findings is provided in Table 2. Quantitatively, six studies primarily evaluated overall HAI outcomes, two primarily assessed device-associated infections, and four primarily examined organism-specific or antimicrobial-resistance-related outcomes.

#### 3.2.1. Overall Healthcare-Associated Infection Outcomes

Six studies primarily evaluated the association between multimodal hand hygiene interventions and overall healthcare-associated infection burden or broad composite infection outcomes. In general, the direction of effect was favorable, although the magnitude and consistency of benefit varied across settings, outcome definitions, and study designs.

In a 383-bed teaching hospital in the United States, Kirkland et al. reported a significant and sustained decline in a hospital-wide healthcare-associated infection index during a 3-year interrupted time-series intervention, with rates falling by 31% from 4.8 to 3.3 per 1000 inpatient-days [38]. Reductions were also observed for *Staphylococcus aureus* infection and bloodstream infection, whereas *Clostridioides difficile* infection remained unchanged. The authors further reported an inverse correlation between monthly hand hygiene compliance and the infection index.

Similarly, Chen et al. found that implementation of the WHO multimodal hand hygiene improvement strategy in a Taiwanese tertiary hospital was associated with a reduction in overall HAI rates from 3.7% to 3.1%, together with significant decreases in urinary tract and respiratory tract infection rates [41]. In Saudi Arabia, Al Kuwaiti et al. also reported favorable changes after a multicomponent WHO-based hand hygiene strategy, with reductions in both overall HAI and CAUTI rates [43].

The longest observation period was reported by Phan et al., who described a sustained, prospective multimodal campaign in a large tertiary obstetrics/gynaecology hospital in Vietnam [45]. Over 9 years, overall HAI incidence density decreased from 1.10 to 0.45 episodes per 1000 patient-days. Significant downward trends were also observed for surgical site infection after gynaecological surgery and for endometritis after abortion, whereas surgical site infection after Caesarean section increased again in the later years of follow-up. The authors explicitly noted, however, that the decrease in HAI other than surgical site infection could only be considered partially attributable to the hand hygiene intervention, because a CAUTI-prevention program implemented in 2012–2013 likely also contributed to the observed decline.

Not all studies showed a clear reduction in overall HAI burden. In medical wards in Spain, Monistrol et al. observed substantial improvement in hand hygiene compliance and alcohol-based hand rub use after a multimodal educational campaign, but overall HAI incidence density remained essentially unchanged (6.93 vs. 6.96 per 1000 hospital-days) [39]. Hospital-acquired MRSA declined numerically, but the difference was not statistically significant. Likewise, Boyce et al. reported that non-*C. difficile* HAIs decreased by 56% after implementation of an automated hand hygiene monitoring system combined with supplementary promotional strategies, but this reduction did not reach statistical significance, while *C. difficile* infections increased by 60%, largely driven by two study units [44].

Taken together, studies evaluating overall HAI outcomes generally suggested a favorable association between multimodal hand hygiene interventions and reduced infection burden at the institutional level. However, the findings were not uniform, and interpretation is tempered by substantial heterogeneity in outcome definitions, follow-up duration, and the presence of co-interventions or broader infection prevention activities occurring alongside hand hygiene promotion.

#### 3.2.2. Device-Associated Infections

Two studies were primarily centered on device-associated infections, and both reported reductions in central line-associated bloodstream infection following multimodal or system-wide hand hygiene improvement efforts. Overall, this subgroup showed some of the clearest favorable signals in the review, although attribution to hand hygiene alone remained limited by concurrent device-related prevention measures and phased implementation.

Johnson et al. described a longitudinal quality improvement project in a 570-bed academic health center in the United States [40]. Over the study period, hospital-wide hand hygiene adherence improved from 58% to 98%, while CLABSI rates decreased from 4.08 to 0.42 per 1000 device-days. However, the intervention was explicitly multifactorial and the program began with hand hygiene education together with implementation of a central line bundle, making it difficult to isolate the specific contribution of hand hygiene improvement to the observed CLABSI reduction.

Shabot et al. evaluated system-wide implementation of the Targeted Solutions Tool across 11 hospitals [42]. Hand hygiene compliance increased from 58.1% during baseline to 95.6% in the final control phase. Over the same period, adult ICU CLABSI rates decreased from 0.83 to 0.42 per 1000 line-days, and ventilator-associated pneumonia rates decreased from 1.04 to 0.57 per 1000 ventilator-days during the improve phase. The authors reported statistically significant associations for most analyses linking study phase or time to lower CLABSI and VAP rates, but also acknowledged that their hospitals had previously implemented CLABSI and VAP bundles, which may have influenced baseline performance.

Additional device-related findings were reported as secondary outcomes in studies primarily focused on overall HAI burden. Al Kuwaiti et al. found a reduction in CAUTI after a WHO-based multicomponent hand hygiene strategy [43], while Chen et al. reported a decrease in bloodstream infection rates [41], although the latter change was smaller than that observed for overall HAI, urinary tract infection, or respiratory tract infection. These findings support a broadly favorable pattern but are better interpreted as secondary supportive evidence rather than as the primary basis for inference on device-associated outcomes.

Overall, the available evidence suggested that multimodal hand hygiene interventions may be associated with lower rates of device-associated infection, especially CLABSI. Nevertheless, the small number of directly relevant studies and the frequent coexistence of central line or ventilator bundles argue for cautious interpretation of causality.

#### 3.2.3. Organism-Specific and Antimicrobial-Resistance-Related Outcomes

Four studies primarily examined organism-specific or antimicrobial-resistance-related outcomes, and together they showed a more heterogeneous pattern than studies assessing overall HAI burden. Favorable associations were reported for some MRSA-, VRE-, and antimicrobial-resistance-related endpoints, but the effects were not consistent across all organisms, settings, or hospitals.

Larson et al. evaluated an organisational climate intervention in two hospitals and found that reductions in MRSA and VRE from baseline to follow-up were greater in the intervention hospital than in the comparison hospital, with the clearest signal seen for VRE [35]. The authors reported that the rate of decrease in VRE was significantly greater in the intervention hospital than in the comparison hospital (85% vs. 44%, *p* < 0.0001), while also noting that outbreaks in the comparison hospital may have influenced the observed differences. Their interpretation was that VRE may have been a particularly sensitive indicator of infection-prevention strategies such as handwashing.

Stone et al. provided the largest-scale evidence in this category through a prospective ecological interrupted time-series evaluation of the national *Cleanyourhands* campaign in England and Wales [37]. Over the four-year study period, MRSA bacteraemia rates fell from 1.88 to 0.91 cases per 10,000 bed-days and *C. difficile* infection fell from 16.75 to 9.49 cases per 10,000 bed-days, whereas MSSA bacteraemia did not fall. Increased procurement of soap was independently associated with reduced *C. difficile* infection, and increased procurement of alcohol hand rub was associated with reduced MRSA bacteraemia in the later phase of the study. At the same time, the authors documented other concurrent national interventions, including the Health Act 2006, the Saving Lives campaign, and Department of Health improvement visits, all of which complicated attribution of effect to hand hygiene promotion alone.

In the multicenter study by Trick et al., a multimodal intervention increased adherence to hand hygiene recommendations across intervention hospitals, but a significant reduction in hospital-acquired antimicrobial-resistant bacteria was seen in only one intervention hospital, namely the hospital that experienced the greatest increase in hand hygiene performance [36]. No comparable decreases were observed in the other intervention hospitals. This study therefore suggested that benefits on antimicrobial-resistance-related outcomes may be achievable, but not uniformly across institutions.

In Spain, Mestre et al. evaluated a two-phase hospital-wide intervention that combined the WHO multimodal hand hygiene strategy with continuous quality improvement methods and statistical process control [46]. Over the intervention period, overall hand hygiene compliance improved from 57% during the preintervention period to 78% in phase 1 and 84% in phase 2, and the authors reported a small but significant decrease in healthcare-acquired MRSA colonization/infection rates over time. The study therefore provided an additional favorable signal for MRSA-related outcomes in the context of an institutional multimodal hand hygiene program. However, as in other single center quasi-experimental quality-improvement studies, the absence of a contemporaneous control group and the possibility of unmeasured confounding limit causal attribution.

Several studies primarily assigned to other outcome categories provided secondary organism-specific findings that reinforced the mixed nature of this evidence. Monistrol et al. reported a numerical decline in new hospital-acquired MRSA after intervention, but without statistical significance [39]. Kirkland et al. found a significant reduction in S. aureus infection, while *C. difficile* remained essentially unchanged [38]. In contrast, Boyce et al. observed an increase in *C. difficile* infections despite a favorable, though non-significant, trend for non-*C. difficile* HAIs [44].

Overall, organism-specific outcomes provided supportive but less consistent evidence than broader HAI endpoints. Reductions were observed for selected organisms, particularly VRE, MRSA bacteraemia, and some hospital-acquired antimicrobial-resistant bacteria, but not across all measured pathogens, and several studies were affected by important co-interventions or context-specific factors that limited causal interpretation.

#### 3.2.4. Structured Analysis of Heterogeneity Across Included Studies

Overall, organism-specific Considerable heterogeneity was present across the included studies and can be understood across several dimensions. First, intervention frameworks differed. Some studies explicitly implemented the WHO multimodal hand hygiene strategy or close variants of it [41,43,45,46], whereas others used locally developed institutional programs emphasizing organisational climate, leadership, accountability, quality-improvement tools, or automated monitoring. Despite these differences, the broad direction of effect was generally similar, with more favorable findings in studies implementing sustained, institution-level multimodal programs than in short or less intensive campaigns.

Second, study duration and implementation intensity varied substantially. Shorter evaluations or studies with relatively brief pre-post comparisons tended to provide less stable estimates and were more vulnerable to contextual fluctuations, whereas longer-running institutional campaigns more often demonstrated sustained directional improvement but also had greater exposure to secular trends and co-interventions over time [38,45,46]. This created a trade-off between duration and interpretability: longer follow-up improved plausibility of sustained implementation effects, but also increased the difficulty of attributing outcome changes specifically to hand hygiene promotion.

Third, the scope and setting of implementation differed. Some interventions were hospital-wide within a single institution, others were implemented across multiple wards, one study was system-wide across 11 hospitals, and one was a national campaign evaluated ecologically at trust level [37,42]. These differences likely contributed to variation in both implementation fidelity and measured outcomes. In general, broader institutional or system-level programs were more likely to show favorable trends in overall HAI or device-associated outcomes, whereas organism-specific outcomes appeared more context dependent.

Fourth, and most importantly for interpretation, the included studies used heterogeneous clinical outcomes and denominators. Outcomes included overall HAI rates, composite institutional indices, CLABSI, CAUTI, VAP, MRSA, VRE, *Clostridioides difficile*, and other antimicrobial-resistance-related endpoints. Denominators also varied substantially and included inpatient-days, hospital-days, patient-days, device-days, admissions, exposure-days, and standardized infection ratios. This variability limited direct comparison of effect magnitude across studies and was one of the principal reasons a quantitative meta-analysis was not undertaken. Taken together, the evidence is more interpretable at the level of directional patterns within outcome families than through cross-study comparison of absolute effect size.

### 3.3. Risk of Bias and Certainty of Evidence

Using ROBINS-I, all 12 included studies were judged to be at either serious or critical overall risk of bias. Ten studies were rated as having serious overall risk of bias, while two were judged to be at critical risk of bias. Across the body of evidence, bias due to confounding was the dominant concern, and this domain was rated as serious or critical in all included studies. This was mainly related to the frequent absence of contemporaneous control groups, the use of uncontrolled before-after or interrupted time-series designs, phased or evolving implementation of interventions, and the presence of important co-interventions or broader infection prevention activities introduced alongside hand hygiene promotion.

A further recurrent concern was bias in the classification of interventions, which was judged as serious in most studies because intervention start points were often not sharply delimited, and implementation commonly occurred in stages rather than as a single clearly defined exposure. By contrast, most studies were judged to be at low risk of bias in the domains of participant selection, deviations from intended interventions, missing data, and outcome measurement, although some individual studies raised concerns in these areas. Bias in selection of the reported result was most often rated as moderate, reflecting the lack of prespecified analytic protocols and the possibility of selective emphasis on particular outcomes, models, or time-point comparisons. Detailed study-level ROBINS-I assessments and supporting justifications are provided in Supplementary File S3.

A GRADE-based summary of certainty of evidence by outcome category is provided in Supplementary Table S3.

## 4. Discussion

### 4.1. Interpretation of Main Findings in the Context of Previous Reviews

This systematic review focused specifically on quasi-experimental evaluations of hospital-wide or multi-ward multimodal hand hygiene interventions in acute care hospitals and synthesized their association with clinical healthcare-associated infection outcomes, rather than with hand hygiene compliance alone. Across the 12 included studies, the overall direction of effect was generally favorable, with most studies reporting reductions in overall HAI burden, selected device-associated infections, or some organism-specific outcomes after implementation of multimodal hand hygiene programs. The clearest and most consistent signals were observed for broader institutional HAI outcomes and for some device-associated infections, particularly CLABSI, whereas organism-specific outcomes such as MRSA, VRE, *Clostridioides difficile*, and broader antimicrobial-resistance-related endpoints were more heterogeneous across studies and settings. These findings should nevertheless be interpreted cautiously, because all included studies were non-randomized and were judged to be at serious or critical overall risk of bias, most commonly due to confounding, phased implementation, lack of contemporaneous controls, and the coexistence of other infection-prevention interventions. Importantly, the review was restricted to studies in which multimodal hand hygiene promotion was the clearly identified intervention focus; however, because institutional hand hygiene programmes are often implemented in complex real-world settings, coexisting infection prevention activities were still common and should be understood as an important limitation of causal interpretation rather than as evidence that the review addressed broad infection prevention bundles more generally.

Our findings are broadly consistent with previous syntheses of the literature which suggest that hand hygiene improvement is associated with lower HAI rates, but they also help refine that literature by addressing a narrower and more clinically focused question. In particular, Mouajou et al. examined the relationship between hand hygiene compliance and HAI incidence and sought to identify a compliance threshold associated with the lowest infection rates, concluding that a negative relationship was observed up to approximately 60% compliance, but that causality could not be inferred [47]. By contrast, the present review did not aim to define an “optimal” compliance target. Instead, it examined whether multimodal institutional hand hygiene programs implemented in acute care hospitals were associated with measurable changes in clinical infection outcomes. Our review is therefore complementary rather than duplicative: it narrows the evidence base to institution-level or multi-ward multimodal interventions and excludes studies limited to process outcomes, highly specialized single-unit settings, long-term care facilities, or broader bundles in which the independent contribution of hand hygiene could not reasonably be interpreted.

Our findings are broadly compatible with other recent reviews, although those reviews addressed different intervention models and outcomes. Zhang et al., for example, found that intelligent hand hygiene technologies were associated with improved compliance and lower healthcare-associated infection rates, but not with a clear reduction in multidrug-resistant organism detection, and emphasized that the overall certainty of evidence was low [48]. Likewise, Doronina et al. concluded that both single and combined interventions can improve hand hygiene compliance, but highlighted the limited methodological robustness and sustainability data in the available literature [49]. Taken together with our results, these reviews suggest that multimodal or technology-supported hand hygiene strategies are plausible and often associated with benefit, but that the clinical literature remains constrained by heterogeneous designs, variable outcome definitions, and difficulty disentangling the independent effect of hand hygiene from that of concurrent infection-prevention measures.

A further interpretive point is that the apparent effectiveness of multimodal hand hygiene programs is likely to depend heavily on context. Issa et al., in a review focused on emergency departments, emphasized that hand hygiene performance is strongly shaped by workload, crowding, physical layout, time pressure, and the unpredictability of clinical workflow, and concluded that improvement strategies must be adapted to the realities of the local environment [50]. Although their review addressed EDs rather than inpatient acute-care hospitals more broadly, the principle is relevant here: the success of multimodal hand hygiene interventions is unlikely to depend only on the nominal intervention components, but also on whether the local setting allows those components to be implemented consistently and sustained over time. This may help explain why some of the included studies showed substantial and durable reductions in HAI, whereas others reported more modest or mixed effects despite apparently similar intervention labels.

Heterogeneity in this review was not limited to setting alone, but also involved intervention framework, duration of implementation, and outcome architecture. WHO-based multimodal programmes and locally developed institutional programmes often shared core components such as education, reminders, audit and feedback, and improved access to alcohol-based hand rub, but differed in implementation intensity, organisational support, and analytic approach. Likewise, longer-running studies were more likely to demonstrate sustained favorable trends, but were also more exposed to secular changes and concurrent infection-prevention measures. Comparisons across studies were further constrained by the use of non-equivalent clinical endpoints and denominators, including patient-days, device-days, admissions, exposure-days, and standardized infection ratios. For this reason, the most meaningful interpretation of the evidence lies in broad directional patterns within outcome categories rather than in direct comparison of apparent effect magnitude across studies.

Abad et al., although studying cohorting rather than hand hygiene specifically, showed that multifaceted infection-control programs may be associated with reduced infection rates even when the independent contribution of a single component cannot be reliably separated [51]. This is directly relevant to the present review, where several studies reported favorable clinical trends but attribution to hand hygiene alone remained difficult because interventions were implemented as part of broader infection-prevention efforts.

Overall, our findings suggest that multimodal hand hygiene programs are most likely to be effective when embedded in a supportive institutional context with visible leadership, regular feedback, accessible products, and sufficient implementation intensity, rather than when delivered as isolated or short-lived campaigns.

### 4.2. Strengths and Limitations of the Evidence and of This Review

Our review was built around prespecified eligibility criteria that deliberately narrowed the scope to acute care hospitals, multimodal hospital-wide or multi-ward interventions, quasi-experimental designs, and clinical infection outcomes rather than process measures alone. This more focused approach distinguishes the present review from broader syntheses such as that of Mouajou et al., which addressed a different question centered on hand hygiene compliance thresholds across a much wider range of study designs and settings [47]. Formal inter-reviewer agreement statistics were not calculated, which may limit the extent to which the reproducibility of the screening process can be quantified.

The main limitation of the underlying evidence is that the available literature remains dominated by non-randomized, quasi-experimental designs conducted in complex real-world hospital environments, where multimodal hand hygiene programs are often introduced alongside other infection prevention and control activities. In our review, this was reflected by the ROBINS-I assessments, in which all included studies were judged to be at serious or critical overall risk of bias, most commonly because of confounding, phased implementation, lack of contemporaneous controls, and difficulty separating the effect of hand hygiene from that of co-interventions. Although studies in which hand hygiene was not the clearly central intervention or in which its independent contribution could not be reasonably interpreted were excluded a priori, residual confounding remained a major limitation of the included literature, reflecting the complexity of real-world infection prevention interventions. This challenge is not unique to the present review. Mouajou et al. similarly concluded that causality could not be inferred from the available observational literature linking hand hygiene compliance to HAI rates [47], while Abad et al., in a different infection-control context, showed that multifaceted programs may be associated with declining infection rates even when the independent contribution of a single component remains difficult to determine [51].

A second limitation of the evidence base is the substantial clinical and methodological heterogeneity across studies. This heterogeneity was also the reason a quantitative meta-analysis was not undertaken, because the included studies did not provide sufficiently comparable interventions, effect measures, outcome definitions, or denominators to support meaningful statistical pooling. The included articles differed in intervention composition, duration of implementation, hospital context, outcome definitions, denominators, surveillance approaches, and analytical methods. Importantly, the studies did not simply measure the same construct in different ways; rather, they often assessed different clinical outcome families altogether, including overall HAI rates, device-associated infections, organism-specific outcomes, and composite institutional indices, using denominators such as patient-days, hospital-days, device-days, admissions, exposure-days, or standardized infection ratios. As a result, the observed effects were more interpretable as within-category directional trends than as directly comparable estimates across the full evidence base, and they were more consistent for broad institutional HAI outcomes than for organism-specific endpoints. Prior reviews have highlighted similar problems. Mouajou et al. described marked heterogeneity in study design, setting, HAI types [47], and reporting, whereas Zhang et al. found that even for technologically enhanced hand hygiene interventions, the overall certainty of evidence for clinical outcomes remained low and important heterogeneity persisted, particularly for multidrug-resistant organism outcomes [48].

A further limitation is the possibility of publication bias, since institutional quality-improvement interventions with favorable or notable results may be more likely to be written up and published than neutral or negative experiences.

### 4.3. Implications for Practice and Future Research

For clinical practice, the present review supports the continued use of multimodal hand hygiene programs as part of institutional infection prevention strategies in acute care hospitals. Across the included studies, the overall direction of effect was generally favorable, particularly for broader HAI outcomes and some device-associated infections, and this pattern is consistent with prior reviews suggesting that hand hygiene interventions are most likely to be effective when they combine education, reminders, monitoring, feedback, accessible hand-rub infrastructure, and visible leadership support. Doronina et al. found that both single and combined interventions can improve hand hygiene compliance, but that more sustained benefits were more likely with multimodal and leadership-directed approaches [49], while Zhang et al. similarly suggested that monitoring and feedback technologies can contribute to improved compliance and lower HAI rates, albeit on a low-certainty evidence base [48]. Taken together, these findings suggest that hospitals should favor sustained, system-level, multimodal implementation over isolated or short-lived campaigns.

However, the review does not support interpreting hand hygiene as a stand-alone solution or assuming that higher compliance will necessarily translate in a linear fashion into lower rates of every HAI outcome. Mouajou et al. were unable to identify a causal compliance threshold from the available literature, and our review likewise found that organism-specific outcomes were more variable than broader HAI measures [47]. In addition, contextual factors are likely to be critical. Issa et al. emphasized that hand hygiene performance is shaped by workload, crowding, physical layout, and workflow complexity, and although their review focused on emergency departments, the broader lesson is relevant to acute care hospitals: implementation strategies must be adapted to local operational realities rather than applied as uniform packages [50].

For future research, there is a clear need for stronger quasi-experimental studies capable of improving causal inference. Controlled before-after studies, well-conducted interrupted time-series designs with clearly defined intervention start points and sufficient pre- and post-intervention measurements, and explicit reporting of co-interventions, denominator definitions, and surveillance methods would materially strengthen the literature. Future studies should also attempt to distinguish the effect of hand hygiene interventions from that of broader infection prevention bundles, since this remains a central interpretive problem in both the current review and related reviews of multifaceted infection-control strategies. Where technology-enhanced monitoring systems are used, studies should move beyond compliance alone and evaluate clinically meaningful outcomes with longer follow-up and better control of confounding.

## 5. Conclusions

Multimodal hand hygiene programs implemented at hospital-wide or multi-ward level in acute care hospitals were generally associated with favorable trends in clinical healthcare-associated infection outcomes, particularly for broader institutional HAI measures and some device-associated infections. However, the evidence base remains limited by non-randomized study designs, substantial clinical and methodological heterogeneity, frequent co-interventions, and overall serious or critical risk of bias across the included studies. The findings therefore support multimodal hand hygiene as an important component of institutional infection prevention strategies, but not as a stand-alone intervention whose independent effect can always be clearly isolated. More rigorous quasi-experimental studies, with clearer intervention boundaries, better control of confounding, and consistent reporting of clinical outcomes, are needed to strengthen causal inference.

## Figures and Tables

**Table 1:** Overall Characteristics of Included Studies. Abbreviations: ABHR, alcohol-based hand rub; CAUTI, catheter-associated urinary tract infection; CLABSI, central line-associated bloodstream infection; HAI, healthcare-associated infection; HH, hand hygiene; ICU, intensive care unit; MRSA, methicillin-resistant *Staphylococcus aureus*; NICU, neonatal intensive care unit; SSI, surgical site infection; VAP, ventilator-associated pneumonia; VRE, vancomycin-resistant *Enterococcus*.

Study ID	Country	Study Design	Setting	Study Period/Duration	Intervention (Key Components)	Comparator/Baseline	Clinical Outcome(s) Assessed
Larson et al., 2010 [35]	USA	Quasi-experimental clinical trial (controlled before–after)	Two ~250-bed hospitals in the Mid-Atlantic region; adult MICU and NICU study units	August 1997–December 1998	Top-down organisational climate intervention based on Schein’s framework; leadership support, education, feedback, role modelling, competency reinforcement	Intervention hospital vs. comparison hospital; baseline, implementation, and follow-up phases	Nosocomial MRSA and VRE infection rates
Trick et al., 2007 [36]	USA	Prospective multicentre controlled interventional observational study	Four hospitals (acute/long-term care, community, public teaching, university teaching), multiple ICUs and wards	October 1999–December 2002	Increased ABHR availability, interactive education, poster campaign in 3 intervention hospitals	Three intervention hospitals vs. one control hospital; pre/post quarterly trends	Incidence of antimicrobial-resistant bacteria from clinical cultures
Stone et al., 2012 [37]	England and Wales	Prospective ecological interrupted time-series study	Acute NHS hospital trusts, national campaign setting	4-year national evaluation	*Cleanyourhands* campaign: bedside alcohol hand rub, posters, regular audit/feedback, patient empowerment materials	Trust-level pre/post time-series trends	MRSA bacteraemia, MSSA bacteraemia, *Clostridium difficile* infection
Kirkland et al., 2012 [38]	USA	Interrupted time series with sequential interventions and 1-year follow-up	383-bed teaching hospital, rural New Hampshire	January 2006–December 2009	Multifaceted initiative targeting leadership/accountability, measurement/feedback, hand sanitiser availability, education/training, marketing/communication	Baseline year and post-intervention follow-up within longitudinal series	Healthcare-associated infection index; *S. aureus* infections; bloodstream infections; *C. difficile* infection
Monistrol et al., 2012 [39]	Spain	before-after intervention study with 1-year follow-up	Three internal medicine wards in a 500-bed tertiary hospital	PRE/POST periods plus 1-year follow-up	Multimodal educational campaign promoting ABHR use; WHO observation methodology	PRE vs. POST intervention periods and follow-up	Overall HAI incidence density; hospital-acquired MRSA incidence density
Johnson et al., 2014 [40]	USA	Descriptive time-series quality improvement project	570-bed academic health centre comprising 7 hospitals and >50 clinics	April 2006–September 2012	Multifactorial action plan focused on staff education, accountability, product selection/accessibility, and organisational culture	Historical baseline before implementation	CLABSI rate
Chen et al., 2016 [41]	Taiwan	Before-and-after interventional study	1408-bed tertiary teaching hospital	January 2010–September 2011	WHO multimodal HH improvement strategy: point-of-care ABHR, tailored education, reminders, evaluation/feedback, institutional safety climate	Pre- vs. post-intervention periods	Overall HAI rate; urinary tract infection; respiratory tract infection; bloodstream infection
Shabot et al., 2016 [42]	USA	System-wide longitudinal quality improvement evaluation	11 hospitals and 150 inpatient units in a large health system	October 2010–December 2014	Targeted Solutions Tool (TST) using DMAIC/Lean/Six Sigma; targeted interventions based on causes of noncompliance	Baseline vs. improve vs. control phases	Adult ICU CLABSI; VAP
Al Kuwaiti et al., 2017 [43]	Saudi Arabia	Prospective interventional before-after study	King Fahd Hospital of the University, inpatient wards	January 2014–December 2016; 12-month intervention	WHO-based multicomponent HH strategy plus management engagement, education, reminders, posters/screensavers, monthly evaluation and feedback	Pre- vs. post-intervention phases	Overall HAI rate; CAUTI rate
Boyce et al., 2019 [44]	USA	Retrospective nonrandomized observational quasi-experimental study	Single 93-bed nonprofit acute-care hospital; 4 nursing units	2014–2018	Automated HH monitoring system plus frontline ownership, leadership support, feedback, supplementary promotional activities, Toyota Kata methodology	Baseline and sequential intervention periods	Non-*C. difficile* HAIs; *C. difficile* infection
Phan et al., 2020 [45]	Vietnam	Prospective quasi-experimental observational study	900-bed maternity/gynaecology-obstetrics tertiary-care centre	2010–2018	WHO multimodal strategy with repeated campaigns, point-of-care ABHR, local ABHR production, patient participation	Longitudinal pre/post trend across campaign period	HAI other than SSI; SSI after gynaecological surgery; endometritis after abortion; SSI after C-section
Mestre et al., 2012 [46]	Spain	Pre-post interventional study with two phases and time-series process analysis	Private 200-bed teaching hospital; eight medical-surgical wards, emergency department, and 11-bed polyvalent ICU	Preintervention March 2007–December 2009; phase 1 January–December 2010; phase 2 January–December 2011	WHO multimodal hand hygiene strategy followed by a continuous quality improvement phase with increased bedside alcohol hand rub placement, more frequent audits (“3/3 strategy”), standardized corrective-action recording, bimonthly feedback using statistical process control charts, and ongoing institutional support	Preintervention baseline vs. phase 1 and phase 2 intervention periods	Healthcare-acquired MRSA colonization/infection rate

**Table 2:** Summary of Clinical Outcomes Associated with Multimodal Hand Hygiene Interventions in Included Studies. Abbreviations: AMR, antimicrobial resistance; CAUTI, catheter-associated urinary tract infection; *C. difficile, Clostridioides difficile*; CLABSI, central line-associated bloodstream infection; HAI, healthcare-associated infection; ICU, intensive care unit; IPC, infection prevention and control; MRSA, methicillin-resistant *Staphylococcus aureus*; MSSA, methicillin-susceptible *Staphylococcus aureus*; SSI, surgical site infection; VAP, ventilator-associated pneumonia; VRE, vancomycin-resistant *Enterococcus*.

Study ID	Primary Clinical Outcome	Outcome Category	Main Finding	Direction of Effect	Statistical Support	Caveat
Larson et al., 2010 [35]	Nosocomial MRSA and VRE infection rates	Organism-specific/AMR-related	Greater reduction in MRSA and VRE in the intervention hospital than in the comparison hospital; clearest signal for VRE	Favorable	Significant for VRE; less clear for MRSA	Non-randomized hospital comparison; outbreaks in comparator hospital may have influenced results
Trick et al., 2007 [36]	Incidence of hospital-acquired antimicrobial-resistant bacteria	Organism-specific/AMR-related	Significant reduction observed in only one intervention hospital, which also had the greatest increase in hand hygiene adherence	Mixed	Significant in one hospital only	No uniform effect across hospitals; clinical control data were limited
Stone et al., 2012 [37]	MRSA bacteraemia, MSSA bacteraemia, *Clostridioides difficile* infection	Organism-specific/AMR-related	MRSA bacteraemia and *C. difficile* decreased over time; MSSA bacteraemia did not	Mixed to favorable	Significant associations reported for some procurement-outcome relationships	Multiple national co-interventions limited attribution to hand hygiene alone
Kirkland et al., 2012 [38]	Hospital-wide healthcare-associated infection index	Overall HAI	HAI index fell from 4.8 to 3.3 per 1000 inpatient-days; *S. aureus* infections also decreased	Favorable	Significant	Multifaceted programme with evolving implementation and broader quality-improvement context
Monistrol et al., 2012 [39]	Overall HAI incidence density	Overall HAI	Overall HAI incidence density remained essentially unchanged after intervention	No clear effect	Not significant	Improved hand hygiene process measures did not translate into measurable overall HAI reduction
Johnson et al., 2014 [40]	CLABSI rate	Device-associated infection	CLABSI declined from 4.08 to 0.42 per 1000 device-days during the programme	Favorable	Clear reduction reported	Central line bundle introduced alongside hand hygiene initiative
Chen et al., 2016 [41]	Overall HAI rate	Overall HAI	Overall HAI decreased from 3.7% to 3.1%; urinary and respiratory tract infections also declined	Favorable	Significant	Before-after design without contemporaneous control; concurrent IPC activities possible
Shabot et al., 2016 [42]	Adult ICU CLABSI rate	Device-associated infection	CLABSI decreased from 0.83 to 0.42 per 1000 line-days; VAP also declined	Favorable	Significant for most phase/time analyses	Prior CLABSI and VAP bundles may have contributed
Al Kuwaiti et al., 2017 [43]	Overall HAI rate	Overall HAI	Overall HAI decreased from 3.37 to 2.59; CAUTI also decreased	Favorable	Reported as significant	Single-centre before-after study without contemporaneous control
Boyce et al., 2019 [44]	Non-*C. difficile* HAI rate	Overall HAI	Non-*C. difficile* HAIs decreased by 56%, but *C. difficile* increased by 60%	Mixed	Reduction in non-*C. difficile* HAIs not statistically significant	Small single-centre study; opposite trend for *C. difficile*
Phan et al., 2020 [45]	HAI other than SSI incidence density	Overall HAI	HAI other than SSI decreased from 1.10 to 0.45 per 1000 patient-days; some procedure-specific infections also declined	Favorable	Significant downward trends reported	CAUTI-prevention programme likely contributed to observed decline
Mestre et al., 2012 [46]	Healthcare-acquired MRSA colonization/infection rate	Organism-specific/AMR-related	A small but significant decrease in healthcare-acquired MRSA coincided with implementation of the hospital-wide WHO/CQI hand hygiene programme; hand hygiene compliance also increased markedly over time	Favorable	Significant	Single-centre pre-post quality-improvement study without contemporaneous control; MRSA outcome may have been influenced by unmeasured confounding

## Data Availability

The original contributions presented in this study are included in the article/Supplementary Material. Further inquiries can be directed to the corresponding author.

## Supplementary Materials

The following supporting information can be downloaded at: https://www.mdpi.com/article/10.3390/jcm15103882/s1, File S1: Full Search Strategies; File S2: Excluded Studies and Reason for Exclusion; File S3: ROBINS-I Risk of Bias Assessment of Included Studies; PRISMA Checklist.

## Abbreviations

The following abbreviations are used in this manuscript:

HAI	Healthcare-associated infection
HH	Hand hygiene
HHC	Hand hygiene compliance
ABHR	Alcohol-based hand rub
CLABSI	Central line-associated bloodstream infection
CAUTI	Catheter-associated urinary tract infection
VAP	Ventilator-associated pneumonia
MRSA	Methicillin-resistant *Staphylococcus aureus*
MSSA	Methicillin-susceptible *Staphylococcus aureus*
VRE	Vancomycin-resistant *Enterococcus*
CDI	*Clostridioides difficile* infection
AMR	Antimicrobial resistance
ICU	Intensive care unit
IPC	Infection prevention and control
ITS	Interrupted time series
ROBINS-I	Risk Of Bias In Non-randomized Studies of Interventions
PRISMA	Preferred Reporting Items for Systematic Reviews and Meta-Analyses
