# Supplementary material for: Multimodal Hand Hygiene Interventions and Clinical Healthcare-Associated Infection Outcomes in Acute Care Hospitals: A Systematic Review of Quasi-Experimental Studies
Source: J Clin Med. 2026 May 18;15(10):3882. doi: 10.3390/jcm15103882 (PMC13207023; doi:10.3390/jcm15103882)
Supplement: Supplementary file 1 [file jcm-15-03882-s001.zip › Supplementary_File_S1_Search_Strategy.pdf]

Multimodal Hand Hygiene Interventions and Clinical Healthcare-Associated Infection Outcomes in Acute Care Hospitals: A Systematic Review of Quasi-Experimental Studies

Supplementary File S1. Full Search Strategies.

The search process was conducted in two stages. An initial search was performed in PubMed and Google Scholar. Before final synthesis, the electronic search was updated and expanded with Embase and Scopus. Full reproducible search strategies for the updated electronic search are provided below. Search dates should be inserted if required by the journal or editorial office.

1. Initial Search Strategy

PubMed and Google Scholar were searched on 12-14 February 2026.

The initial PubMed search combined MeSH terms with free-text title/abstract keywords. The MeSH component included terms related to hand hygiene, cross infection, and hospitals, while the free-text component included synonyms and design-related keywords such as “hand hygiene,” “hand washing,” “healthcare-associated infection,” “hospital-acquired infection,” “nosocomial infection,” “acute care,” “quasi-experimental,” “before and after,” and “pre-post,” as well as specific HAI outcomes including CLABSI, CAUTI, VAP, Clostridioides difficile, MRSA, and VRE.

Google Scholar was used as a supplementary search source using combinations of terms related to hand hygiene, healthcare-associated infections, acute care hospitals, and quasi-experimental interventions. The exact original Google Scholar syntax was not preserved in the initial search record.

Backward reference screening (“snowballing”) was used as a supplementary approach to identify additional relevant reports.

The exact MeSH syntax in PubMed was:

```
("Hand Hygiene"[Mesh]
  OR "Hand Disinfection"[Mesh]
  OR "hand hygiene"[tiab]
  OR "hand washing"[tiab]
  OR handwashing[tiab]
  OR "hand disinfection"[tiab])
AND
("Cross Infection"[Mesh]
  OR "healthcare-associated infection"[tiab]
  OR "health care-associated infection"[tiab]
  OR "hospital-acquired infection"[tiab]
  OR "hospital acquired infection"[tiab]
  OR "nosocomial infection"[tiab]
  OR CLABSI[tiab]
  OR "central line-associated bloodstream infection"[tiab]
  OR CAUTI[tiab]
  OR "catheter-associated urinary tract infection"[tiab]
  OR VAP[tiab]
  OR "ventilator-associated pneumonia"[tiab]
```

OR "Clostridioides difficile"[tiab]
OR MRSA[tiab]
OR VRE[tiab])
AND
("interrupted time series"[tiab]
OR "before and after"[tiab]
OR "before-after"[tiab]
OR "controlled before-after"[tiab]
OR "controlled before and after"[tiab]
OR "quasi experimental"[tiab]
OR "quasi-experimental"[tiab]
OR quasiexperiment*[tiab]
OR "pre post"[tiab]
OR "pre-post"[tiab]
OR pretest-posttest[tiab])
AND
("Hospitals"[Mesh]
OR hospital*[tiab]
OR "acute care"[tiab])
NOT
("nursing home"[tiab]
OR "nursing homes"[tiab]
OR "long term care"[tiab]
OR "long-term care"[tiab])

2. Expanded Search Strategy

The original PubMed search strategy was rerun in PubMed between 8 and 10 May 2026, and the search was expanded to include Embase and Scopus. Table S1 presents the number of records retrieved from each database and the exact search queries used.

Table S1. Database Search Syntax.

Search Engine	Number of Records Found	Exact Search Query
Pubmed	351	("Hand Hygiene"[Mesh]<br>OR "Hand Disinfection"[Mesh]<br>OR "hand hygiene"[tiab]<br>OR "hand washing"[tiab]<br>OR handwashing[tiab]<br>OR "hand disinfection"[tiab])<br>AND<br>("Cross Infection"[Mesh]<br>OR "healthcare-associated infection"[tiab]<br>OR "health care-associated infection"[tiab]<br>OR "hospital-acquired infection"[tiab]<br>OR "hospital acquired infection"[tiab]<br>OR "nosocomial infection"[tiab]<br>OR CLABSI[tiab]<br>OR "central line-associated bloodstream infection"[tiab]

		OR CAUTI[tiab]<br>OR "catheter-associated urinary tract infection"[tiab]<br>OR VAP[tiab]<br>OR "ventilator-associated pneumonia"[tiab]<br>OR "Clostridioides difficile"[tiab]<br>OR MRSA[tiab]<br>OR VRE[tiab])<br>AND<br>("interrupted time series"[tiab]<br>OR "before and after"[tiab]<br>OR "before-after"[tiab]<br>OR "controlled before-after"[tiab]<br>OR "controlled before and after"[tiab]<br>OR "quasi experimental"[tiab]<br>OR "quasi-experimental"[tiab]<br>OR quasiexperiment*[tiab]<br>OR "pre post"[tiab]<br>OR "pre-post"[tiab]<br>OR pretest-posttest[tiab])<br>AND<br>("Hospitals"[Mesh]<br>OR hospital*[tiab]<br>OR "acute care"[tiab])<br>NOT<br>("nursing home"[tiab]<br>OR "nursing homes"[tiab]<br>OR "long term care"[tiab]<br>OR "long-term care"[tiab])
Scopus	442	TITLE-ABS-KEY(<br>("hand hygiene" OR "hand washing" OR handwashing OR "hand disinfection")<br>AND<br>("healthcare-associated infection" OR "health care-associated infection" OR "hospital-acquired infection" OR "hospital acquired infection" OR "nosocomial infection" OR CLABSI OR "central line-associated bloodstream infection" OR CAUTI OR "catheter-associated urinary tract infection" OR VAP OR "ventilator-associated pneumonia" OR "Clostridioides difficile" OR MRSA OR VRE)<br>AND<br>(hospital* OR "acute care")<br>AND<br>("interrupted time series" OR "before and after" OR "before-after" OR "controlled before-after" OR "controlled before and after" OR "quasi experimental" OR "quasi-experimental" OR quasiexperiment* OR "pre post" OR "pre-post" OR pretest-posttest)<br>)<br>AND NOT TITLE-ABS-KEY(<br>"nursing home" OR "nursing homes" OR "long term care" OR "long-term care"<br>)
Embase	282	(<br>'hand hygiene':ti,ab,kw<br>OR 'hand washing':ti,ab,kw<br>OR handwashing:ti,ab,kw<br>OR 'hand disinfection':ti,ab,kw<br>)

		AND<br>(<br>'healthcare-associated infection':ti,ab,kw<br>OR 'health care-associated infection':ti,ab,kw<br>OR 'hospital-acquired infection':ti,ab,kw<br>OR 'hospital acquired infection':ti,ab,kw<br>OR 'nosocomial infection':ti,ab,kw<br>OR clabsi:ti,ab,kw<br>OR 'central line-associated bloodstream infection':ti,ab,kw<br>OR cauti:ti,ab,kw<br>OR 'catheter-associated urinary tract infection':ti,ab,kw<br>OR vap:ti,ab,kw<br>OR 'ventilator-associated pneumonia':ti,ab,kw<br>OR 'clostridioides difficile':ti,ab,kw<br>OR mrsa:ti,ab,kw<br>OR vre:ti,ab,kw<br>)<br>AND<br>(<br>'interrupted time series':ti,ab,kw<br>OR 'before and after':ti,ab,kw<br>OR 'before-after':ti,ab,kw<br>OR 'controlled before-after':ti,ab,kw<br>OR 'controlled before and after':ti,ab,kw<br>OR 'quasi experimental':ti,ab,kw<br>OR 'quasi-experimental':ti,ab,kw<br>OR quasiexperiment*:ti,ab,kw<br>OR 'pre post':ti,ab,kw<br>OR 'pre-post':ti,ab,kw<br>OR pretest-posttest:ti,ab,kw<br>)<br>AND<br>(<br>hospital*:ti,ab,kw<br>OR 'acute care':ti,ab,kw<br>)<br>NOT<br>(<br>'nursing home':ti,ab,kw<br>OR 'nursing homes':ti,ab,kw<br>OR 'long term care':ti,ab,kw<br>OR 'long-term care':ti,ab,kw<br>)
