# Supplementary material for: Multimodal Hand Hygiene Interventions and Clinical Healthcare-Associated Infection Outcomes in Acute Care Hospitals: A Systematic Review of Quasi-Experimental Studies
Source: J Clin Med. 2026 May 18;15(10):3882. doi: 10.3390/jcm15103882 (PMC13207023; doi:10.3390/jcm15103882)
Supplement: Supplementary file 1 [file jcm-15-03882-s001.zip › Supplementary_File_S2_Excluded_Articles.pdf]

Multimodal Hand Hygiene Interventions and Clinical Healthcare-Associated Infection Outcomes in Acute Care Hospitals: A Systematic Review of Quasi-Experimental Studies

Supplementary File S2. Excluded Studies and Reason for Exclusion.

	Reason for exclusion
Larson 2007 [14]	Wrong study design / no clearly defined multimodal intervention; evaluated dissemination and uptake of guidelines across hospitals rather than a clearly defined quasi-experimental multimodal hand hygiene intervention program.
Yue & Pan 2025 [15]	Wrong setting / insufficiently aligned quasi-experimental structure; conducted in a geriatric hospital with a long-term dynamic intervention and no clearly defined intervention point or comparator.
van der Kooi 2022 [16]	Wrong setting; wrong outcome; intensive care unit-only study focusing on individual hand hygiene compliance, without clinical healthcare-associated infection outcomes for the present analysis.
Ragusa 2018 [17]	No clearly defined intervention; correlational surveillance study assessing the relationship between hand hygiene compliance and *Clostridioides difficile* infection rather than a quasi-experimental intervention study.
Lenz 2018 [18]	Wrong setting; pediatric intensive care unit-only study evaluating a catheter infection prevention bundle rather than a hospital-wide or multi-ward hand hygiene intervention.
Ojanperä 2022 [19]	No clearly defined multimodal intervention / observational design; longitudinal observational monitoring study without a discrete intervention point or clearly defined hand hygiene improvement program.
von Lengerke 2019 [20]	Wrong study design; wrong setting; cluster-randomized controlled trial conducted in intensive care and hematopoietic stem cell transplantation units only.
Ebnöther 2008 [21]	Hand hygiene not the clearly central intervention; broad multicomponent infection control program including hand hygiene, antibiotic prophylaxis changes, isolation, and infectious diseases consultation, preventing attribution of effect specifically to hand hygiene.
Harne-Britner 2011[22]	Insufficient intervention scope; small unit-level study conducted in three medical-surgical units, primarily focused on nursing behavior change rather than a hospital-wide or broad multi-ward hand hygiene program.
Le 2007 [23]	Wrong setting / single-unit study; intervention restricted to a single neurosurgical ward with one ward control, not hospital-wide or clearly multi-ward.
Koff 2011[24]	Wrong setting; single intensive care unit study, with coexisting ventilator and central line bundles.

Schelenz 2005 [25]	Hand hygiene not the clearly central intervention; highly specialized cardiothoracic unit program including screening, isolation, decolonization, antibiotic changes, environmental cleaning, and hand hygiene.
Gilbert 2020 [26]	Insufficient intervention scope / substantial confounding; practice-improvement study limited to two surgical wards, with major concurrent environmental decontamination measures.
Stone 1998 [27]	Hand hygiene not the clearly central intervention; infection control policy combined handwashing with antibiotic restriction and feedback, making the specific contribution of hand hygiene inseparable.
Salama 2013 [28]	Wrong setting; study conducted exclusively in a single adult medical-surgical intensive care unit.
Mishal 2001 [29]	Hand hygiene not the clearly central intervention; program centered on contact isolation precautions for methicillin-resistant *Staphylococcus aureus*, with handwashing as one component rather than the principal intervention.
Kong 2021 [30]	Hand hygiene not the clearly central intervention / major co-interventions; study evaluated COVID-era hospital-wide infection prevention measures including hand hygiene, universal masking, expanded environmental services, and visitor restrictions, making the independent contribution of hand hygiene inseparable.
dos Santos 2011 [31]	Hand hygiene not the clearly central intervention; before-after study primarily evaluated antimicrobial stewardship and carbapenem resistance, with alcohol-based hand rub consumption analysed as a covariate rather than as a defined multimodal hand hygiene intervention.
Lee 2009 [32]	Hand hygiene not the clearly central intervention; institution-wide education study was embedded within an established MRSA control programme including screening and additional precautions, and did not evaluate a distinct multimodal hand hygiene programme as the principal intervention.
Farhoudi 2016 [33]	Wrong outcome; quasi-experimental WHO-based hand hygiene programme reported hand hygiene compliance improvement but did not report clinical healthcare-associated infection outcomes.
Cheng 2014 [34]	Hand hygiene not the clearly central intervention / broader phased infection-control bundle; although a hospital-wide hand hygiene campaign was implemented, the overall programme also included contact precautions, dedicated equipment, and chlorhexidine bathing, preventing clear attribution of effect specifically to hand hygiene.
