# Supplementary material for: Multimodal Hand Hygiene Interventions and Clinical Healthcare-Associated Infection Outcomes in Acute Care Hospitals: A Systematic Review of Quasi-Experimental Studies
Source: J Clin Med. 2026 May 18;15(10):3882. doi: 10.3390/jcm15103882 (PMC13207023; doi:10.3390/jcm15103882)
Supplement: Supplementary file 1 [file jcm-15-03882-s001.zip › Supplementary_File_S3_RoB.pdf]

Multimodal Hand Hygiene Interventions and Clinical Healthcare-Associated Infection Outcomes in Acute Care Hospitals: A Systematic Review of Quasi-Experimental Studies

Supplementary File 3. ROBINS-I Risk of Bias Assessment of Included Studies.

Assessment method

Risk of bias was assessed with the original ROBINS-I tool (Risk Of Bias In Non-randomised Studies of Interventions) for the key clinical outcome selected from each included study. Assessments were performed independently by two reviewers and finalized by consensus. The effect of interest was the effect of implementation of the multimodal hand hygiene program rather than the effect of perfect adherence. Judgements were made for the seven standard ROBINS-I domains and categorized as low, moderate, serious, critical, or no information. Overall risk of bias was set at least as severe as the most severe domain and was escalated when the pattern of domain-level concerns justified a higher overall judgement.

Operationalization of ROBINS-I in this review

**Target trial**	An acute care hospital, or multiple wards within an acute care hospital, implementing a hospital-wide or multi-ward multimodal hand hygiene programme compared with usual practice, an earlier pre-intervention period, or a contemporaneous comparator, with the same clinical HAI outcome definitions and surveillance framework.
**Unit of assessment**	Outcome level. One key clinical infection outcome was assessed for each included study result presented in this supplement.
**Effect of interest**	The effect of assignment or implementation of the hand hygiene programme, not the effect of complete adherence by individual healthcare workers.
**Prespecified confounders and co-interventions**	Secular trends in HAI incidence; outbreaks; case-mix and severity; staffing levels, workload, and crowding; device utilization; antimicrobial stewardship; environmental cleaning or disinfection changes; screening, isolation, or contact precautions; changes in microbiology practice or HAI surveillance definitions; and concurrent IPC bundles or safety initiatives.
**Interpretation rule**	Results judged at critical risk of bias were considered highly limited for causal inference. In this review, confounding and intervention classification were anticipated to be the dominant sources of bias because most studies used quasi-experimental pre-post or interrupted time series designs without a contemporaneous untreated control.

Table S2. Final ROBINS-I assessments for included studies.

Study	Outcome assessed	D1 Conf.	D2 Sel.	D3 Class.	D4 Dev.	D5 Miss.	D6 Meas.	D7 Report	Overall	Support for final judgement
Johnson et al. 2014 [40]	CLABSI rate per 1,000 device-days	Critical	Low	Serious	Low	Low	Low	Moderate	Critical	Critical confounding in an uncontrolled phased QI programme with a concurrent central line bundle and no contemporaneous control; the CLABSI reduction cannot be attributed confidently to hand hygiene alone.
Larson et al. 2010 [35]	VRE nosocomial infection rate per 1,000 patient-care days	Serious	Low	Serious	Moderate	Low	Low	Moderate	Serious	Serious confounding and intervention misclassification in a convenience-selected two-hospital comparison with early contamination of baseline and outbreaks in the comparator hospital.
Stone et al. 2012 [37]	Hospital-acquired MRSA bacteraemia rate per 10,000 bed days	Serious	Moderate	Moderate	Low	Moderate	Low	Moderate	Serious	Serious residual confounding remained because the national campaign had no untreated comparator and overlapped with multiple MRSA-focused policies despite statistical adjustment.
Kirkland et al. 2012 [38]	Healthcare-associated infection index rate per 1,000 inpatient days	Serious	Low	Serious	Low	Moderate	Low	Moderate	Serious	Serious bias from uncontrolled sequential implementation and residual time-varying confounding; broader safety-culture and quality-improvement changes may also explain lower infection rates.
Al Kuwaiti 2017 [43]	Hospital-acquired infection (HAI) rate per 1,000 patient-days	Serious	Low	Serious	Low	Low	Moderate	Moderate	Serious	Uncontrolled before-after design with no concurrent control or adjustment for major time-varying confounders, and the intervention periods were not sharply defined.
Boyce et al. 2019 [44]	Non-CDI healthcare-associated infection rate per 10,000 patient-days (pooled across 4 study units)	Serious	Low	Serious	Low	Low	Low	Moderate	Serious	Single-hospital quasi-experimental design without a formal control, with stepwise implementation and sequential promotional components that limit causal attribution.
Chen et al., 2016 [41]	Overall healthcare-associated infection (HAI) rate per 1,000 admission patient days	Serious	Low	Serious	Low	Low	Low	Moderate	Serious	Uncontrolled before-after study with overlapping baseline and implementation periods and no adjustment for important time-varying confounders or coexisting IPC policies.
Trick et al., 2007 [36]	Overall incidence of hospital-acquired antimicrobial-resistant bacteria among clinical isolates per 1,000 patient-days (hospital A)	Serious	Low	Moderate	Low	Low	Low	Moderate	Serious	Clinical outcome analysis lacked a comparable concurrent control, and a separate antimicrobial-prescribing intervention and other unmeasured changes could have confounded the association.
Phan et al., 2020 [45]	Healthcare-associated infection (HAI) other than surgical-site infection incidence density per 1,000 patient-days	Critical	Low	Serious	Low	Low	Low	Moderate	Critical	Critical confounding in a 9-year uncontrolled study with explicit co-intervention from a CAUTI-prevention programme; the non-SSI HAI decline cannot be separated reliably from other changes.
Monistrol et al. 2012 [39]	Overall hospital-acquired infection (HAI) incidence density per 1,000 hospital-days	Serious	Low	Moderate	Low	Low	Low	Moderate	Serious	Serious bias from an uncontrolled pre-post design without risk-adjusted analysis, despite similar measured baseline characteristics in the selected wards.

Study	Outcome assessed	D1 Conf.	D2 Sel.	D3 Class.	D4 Dev.	D5 Miss.	D6 Meas.	D7 Report	Overall	Support for final judgement
Shabot et al. 2016 [42]	Adult ICU CLABSI rate per 1,000 central line-days	Serious	Low	Serious	Low	Low	Low	Moderate	Serious	Residual confounding in an uncontrolled system-wide interrupted time series and uncertainty in intervention start dates, despite standardized outcome measurement.
Mestre et al. 2012 [46]	Healthcare-acquired MRSA colonization/infection rate per patient-days	Serious	Low	Serious	Low	Low	Low	Moderate	Serious	Serious confounding and intervention misclassification in a single-centre pre-post quality-improvement study with a two-phase evolving intervention, no contemporaneous control, and potential influence of unmeasured institutional factors on MRSA trends.

Abbreviations: Conf., confounding; Sel., selection of participants; Class., classification of intervention; Dev., deviations from intended interventions; Miss., missing data; Meas., measurement of outcomes; Report, selection of the reported result; HAI, healthcare-associated infection; HH, hand hygiene; CLABSI, central line-associated bloodstream infection; CDI, Clostridioides difficile infection; MRSA, methicillin-resistant Staphylococcus aureus; VRE, vancomycin-resistant Enterococcus.

Table S3. GRADE Assessment of Certainty of Evidence by Outcome Category.

Outcome category	Included studies	Study design	Risk of bias	Inconsistency	Indirectness	Imprecision	Publication bias	Overall certainty of evidence	Rationale for rating
**Overall healthcare-associated infection outcomes**	6 studies [38,39,41,43,44,45]	Non-randomized quasi-experimental studies	Serious	Not serious	Not serious	Not serious	Undetected	Low	All contributing studies were non-randomized and mostly uncontrolled before-after or interrupted time-series evaluations. Risk of bias was serious overall, with one critically biased long-term study and frequent confounding by co-interventions. Effects were generally favorable but not fully consistent across studies, outcome definitions, denominators, and follow-up periods.
**Device-associated infection outcomes**	2 primary studies [40,42]	Non-randomized quasi-experimental studies	Serious	Not serious	Serious	Serious	Undetected	Very low	The direction of effect was generally favorable, especially for CLABSI, but the evidence base was small and strongly limited by non-randomized designs, lack of contemporaneous controls, and major co-interventions such as central-line or ventilator bundles. The small number of directly relevant studies and limited precision reduce confidence in the estimate.
**Organism-specific / antimicrobial-resistance-related outcomes**	4 studies [35,36,37,47]	Non-randomized quasi-experimental studies	Serious	Serious	Not serious	Not serious	Undetected	Low	Although several studies suggested favorable effects for selected outcomes such as MRSA or VRE, the findings were heterogeneous across organisms, settings, and analytic approaches. Important co-interventions, ecological designs, comparator instability, and non-commensurable outcome definitions substantially limit certainty.

Abbreviations: CLABSI, central line-associated bloodstream infection; HAI, healthcare-associated infection.

Explanation: Certainty of evidence was assessed using the GRADE approach at the level of major outcome categories rather than individual study outcomes. Judgments were informed by the ROBINS-I assessments, study design, consistency of findings, clinical and methodological directness, and precision of the available evidence. Because all included studies were non-randomized and most were judged at serious or critical risk of bias, the certainty of evidence was downgraded accordingly.
